# VarI-COSI 2018: a forum for research advances in variant interpretation and diagnostics

**DOI:** 10.1186/s12864-019-5862-3

**Published:** 2019-07-16

**Authors:** Yana Bromberg, Emidio Capriotti, Hannah Carter

**Affiliations:** 1 Department of Biochemistry and Microbiology, Lipman Hall 218, Rutgers University, New Brunswick, NJ 08901 USA; 2 Department of Genetics, Lipman Hall 218, Rutgers University, New Brunswick, NJ 08901 USA; 3 Department of Pharmacy and Biotechnology (FaBiT), University of Bologna, 40126 Bologna, Italy; 4 Division of Medical Genetics, Department of Medicine, University of California, San Diego, CA 92093 USA

## Introduction

Technological advances and decreasing costs of DNA sequencing have created a deluge of genomic data [1–3]. These include millions of genetic variants that require interpretation relative to various traits or diseases. The **Var**iant **I**nterpretation **C**ommunity **O**f **S**pecial **I**nterest **G**roup (VarI-COSI, formerly VarI-SIG) provides an international forum for researchers to share progress in their work to address the need for strategies to detect, annotate and interpret variants in the context of health and disease. The 8th edition of the VarI-COSI meeting [4–9] was held on July 8th, 2018 at the ISMB meeting in Chicago, Illinois (USA).

This year’s meeting of the VarI-COSI featured three keynote talks, 11 research presentations and two industry presentations describing new datasets, bioinformatic methods, and scientific studies aimed at advancing our understanding of genetic variation. In addition, we heard presentations from our sponsors, Variantyx and Qiagen describing technological developments for variant detection and prioritization.

This year, the VarI-COSI was divided into a morning and afternoon session. The morning session began with a keynote by Bonnie Berger describing the potential of genomic crowdsourcing with privacy to significantly boost data availability for genomic studies. Talks in this session described approaches to mine position-specific information from the genome to better annotate and study genetic variation. Speakers described using information about protein structure to study variants at protein interaction interfaces, identifying positions involved in the functional tuning of proteins, using signatures of purifying selection to implicate non-coding Mendelian variants, and annotating variants with an expanded set of conservation categories using machine learning.

The afternoon included two additional keynotes. Mona Singh described work studying variation that affects protein interactions with target molecules to gain insights into the pathogenesis of cancer. Olga Troyanskaya described the application of deep learning to predict the effect of single nucleotide variants on the expression of nearby genes. Talks in the afternoon session included statistical developments to improve meta-analysis in GWAS studies in the presence of heterogeneity, new methods for detecting splicing and structural variants, and different approaches using molecular measurements or diverse phenotypes to gain improved insight into the role of genetic variation in disease processes.

We welcome Antonio Rausell from the Institut Imagine, Paris (France) to the VarICOSI organizing committee.

The VarI-COSI executive committee invites the community to provide feedback regarding meeting content and format, as well as to participate in future sessions of the meeting.

## Manuscript submission and review

For this year’s VarI-COSI special issue received 12 manuscript submissions. All manuscripts were evaluated by at least two reviewers, selected by a panel of three editors and 25 experts in the field (see *Acknowledgements*). After 2 rounds of review 11 manuscripts were accepted for publication. These presented novel strategies for implicating biological functions underlying diverse human phenotypes [10], detecting new genes associated with cardiovascular disease [11], inferring the impact of variants on protein stability [12], predicting the effects of variants in membrane proteins [13], at post-translational modification sites [14], on mRNA translation [15] and on DNA ionization [16].

Other manuscripts describe the development methods for improving variant calling [17] and analysis of structural variants [18] and studying the effects of somatic mutations in cancer on metabolism [19] and tumor interaction with the immune system [20].

The complete program of VarI-COSI meeting 2018 with presentation and poster abstracts is available at http://varicosi.biofold.org/2018/schedu.html .

## Further developments

We are working to organize the next VarI-COSI meeting (ISMB/ECCB 2019; Basel, Switzerland; July 24th, 2019). Further information about this coming meeting is available on our website (http://varicosi.biofold.org/).

## Data Availability

No data are associated to this manuscript.
